# Mechanical forces regulate endothelial-to-mesenchymal transition and atherosclerosis via an Alk5-Shc mechanotransduction pathway

**DOI:** 10.1126/sciadv.abg5060

**Published:** 2021-07-09

**Authors:** Vedanta Mehta, Kar-Lai Pang, Christopher S. Givens, Zhongming Chen, Jianhua Huang, Daniel T. Sweet, Hanjoong Jo, John S. Reader, Ellie Tzima

**Affiliations:** 1 Cardiovascular Medicine, Radcliffe Department of Medicine, University of Oxford, Oxford, UK.; 2 Wellcome Centre for Human Genetics, University of Oxford, Oxford, UK.; 3 McAllister Heart Institute, University of North Carolina at Chapel Hill, Chapel Hill, NC, USA.; 4 Coulter Department of Biomedical Engineering, Emory University and Georgia Institute of Technology, Atlanta, GA, USA.

## Abstract

The response of endothelial cells to mechanical forces is a critical determinant of vascular health. Vascular pathologies, such as atherosclerosis, characterized by abnormal mechanical forces are frequently accompanied by endothelial-to-mesenchymal transition (EndMT). However, how forces affect the mechanotransduction pathways controlling cellular plasticity, inflammation, and, ultimately, vessel pathology is poorly understood. Here, we identify a mechanoreceptor that is sui generis for EndMT and unveil a molecular Alk5-Shc pathway that leads to EndMT and atherosclerosis. Depletion of Alk5 abrogates shear stress–induced EndMT responses, and genetic targeting of endothelial Shc reduces EndMT and atherosclerosis in areas of disturbed flow. Tensional force and reconstitution experiments reveal a mechanosensory function for Alk5 in EndMT signaling that is unique and independent of other mechanosensors. Our findings are of fundamental importance for understanding how mechanical forces regulate biochemical signaling, cell plasticity, and vascular disease.

## Teaser

Mechanical forces regulate endothelial-to-mesenchymal transition via Alk5-Shc pathway.

## INTRODUCTION

Mechanical forces in the vascular system are critical determinants of homeostasis but can also be instigators of disease (*1*). Geometrical variations in arteries determine blood flow patterns and propensity to disease, such that regions with flow disturbances exhibit endothelial cell (EC) dysfunction, inflammation, and lipid accumulation that drive atherosclerosis (*2*, *3*). The endothelium senses biomechanical forces via mechanosensors (*4*) expressed on the cell surface that initiate a complex network of signal transduction pathways that, ultimately, regulate cell and organ function.

A process that has recently been identified to be regulated by mechanical forces is endothelial-to-mesenchymal transition (EndMT). Although the multifunctional cytokine transforming growth factor–β (TGFβ) is a key player in EndMT (*5*–*7*), recent studies have reported that application of atheroprone (or oscillatory/disturbed) shear stress to ECs in vitro and in vivo stimulates the expression of EndMT transcription factors and mesenchymal markers (*8*–*10*). In addition to its role in embryonic development, EndMT has also been implicated in inflammatory cardiovascular diseases (*11*, *12*), including atherosclerosis (*9*, *10*, *13*, *14*). The TGFβ-Alk5 pathway is important for the shear stress response (*15*–*17*), and recent work has shown that endothelial-specific deletion of both *TGFβR1* (*Alk5*) and *TGFβR2* delayed atherosclerotic plaque growth and induced regression of fully established lesions, thus clearly establishing a causal relationship between EndMT and atherosclerosis (*18*). This body of work gives credence to the idea that disturbed shear stress promotes EndMT and contributes to atherogenesis; however, the mechanoreceptor responsible for flow-induced EndMT remains undiscovered. Furthermore, our understanding of the molecular mechanisms by which mechanical forces are decoded into signals that ultimately regulate EndMT and atherosclerosis are underexplored. Last, a direct connection between force, mechanosensing, and EndMT signaling, which ultimately regulates cellular plasticity, has never been demonstrated.

The Src homology and collagen (Shc) is a prototypical adaptor protein that constitutes an essential element of signal propagation as it transduces extracellular signals into intracellular pathways (*19*–*21*). In response to mechanical force, Shc associates with cell-cell and cell matrix adhesions, the two mechanical hotspots within ECs, and activates shear stress responses in vitro and in vivo (*22*, *23*). Given the emerging connections between mechanotransduction, EndMT, and endothelial activation, we investigated the molecular mechanisms by which shear stress regulates EndMT and atherosclerosis. Here, we identify Alk5 as the receptor responsible for mechano-EndMT and establish Shc as a critical downstream driver of EndMT and atherosclerosis in areas of disturbed shear stress.

## RESULTS

### Alk5 is required for flow-induced EndMT

**Fig. 1:**
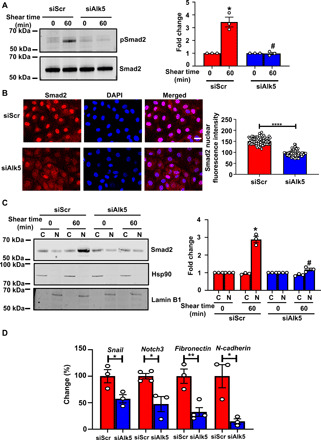
Shear stress induces EndMT via Alk5. (**A**) Mouse ECs were transfected with scrambled (Scr) or *Alk5* siRNA and exposed to fluid shear stress (12 dynes cm^−2^) using a parallel plate system for 60 min. Phosphorylation of Smad2 was determined by Western blotting and quantified using Image Studio Lite v.5.2. (**B**) BAECs were transfected with Scr or *Alk5* siRNA and exposed to fluid shear stress (12 dynes cm^−2^) using a parallel plate system for 60 min. Nuclear translocation of Smad2 was determined by immunofluorescence staining and quantified as mean fluorescence intensity on ImageJ. DAPI, 4′,6-diamidino-2-phenylindole. (**C**) Mouse ECs were transfected with Scr or *Alk5* siRNA and exposed to fluid shear stress (12 dynes cm^−2^) using a parallel plate system for 60 min. The cells were then separated into cytosolic and nuclear fractions before analysis by Western blotting with the Smad2 antibody. Western blots, performed using antibodies against the cytoplasmic protein Hsp90 and nuclear protein Lamin B1, indicated that the extracts are virtually free from cross-contamination. Hsp90 served as the loading control for cytoplasmic extracts and Lamin B1 for nuclear extracts. (**D**) Mouse ECs were transfected with either Scr or *Alk5* siRNA and exposed to atheroprone flow for 48 hours using a cone-and-plate viscometer. Quantitative polymerase chain reaction (qPCR) was performed to quantify the expression of the EndMT markers *Snail*, *fibronectin*, *Notch3*, and *N-cadherin*. Scale bar, 20 μm; *n* = 4. Data are presented as means ± SEM. *P* values were obtained by two-tailed Student’s *t* tests using GraphPad Prism. Phosphorylated are indicated by “p-.“ *P < 0.05, **P < 0.01, ****P < 0.001, and #*P* < 0.05 relative to the respective shear time point of Scr siRNA.

Disturbed shear stress activates EndMT (*8*–*10*); however, the receptor responsible for flow-induced EndMT is unknown. TGFβ induces EndMT via its receptor Alk5, which, upon phosphorylation by its partner TGFβR2, allows the activation of receptor-regulated Smads (i.e., Smad2/3) (*24*). We hypothesized that Alk5 plays a role in EndMT induced by shear stress and adopted a loss-of-function approach by small interfering RNA (siRNA)–mediated transfection to test the role of Alk5 in the endothelium. After confirming efficiency of knockdown of Alk5 using three different siRNAs (fig. S1A), we investigated the direct role of Alk5 in shear stress–induced activation of EndMT using several complementary approaches. First, we subjected ECs transfected with control and Alk5 siRNAs to acute shear stress [which elicits the same sustained signaling observed with chronic disturbed shear stress (*1*)] and examined phosphorylation of Smad2. Alk5-depleted ECs displayed reduced flow-induced phosphorylation of Smad2 compared to Alk5-expressing cells (Fig. 1A and fig. S2). We confirmed the requirement for Alk5 in phosphorylation of Smad2 after exposure of ECs to chronic oscillatory shear stress (fig. S3A). Second, we subjected ECs to the same acute and chronic oscillatory shear stress protocols and examined nuclear translocation of Smad2. Alk5 siRNA–transfected cells showed a significant reduction in nuclear translocation of Smad2 (Fig. 1B and fig. S3B). To corroborate these findings further, we used a third approach that involved cellular fractionation and separation of nuclear versus cytoplasmic fractions. As shown in Fig. 1C, exposure of ECs to shear stress resulted in increased levels of total Smad2 in the nuclear fraction, which was decreased in Alk5-depleted ECs. Fourth, we also examined expression levels of EndMT genes in ECs subjected to chronic oscillatory shear stress. Compared with controls, Alk5 knockdown cells showed significantly less messenger RNA (mRNA) levels of the EndMT transcription factor *Snail* (Fig. 1D), which is involved in up-regulation of mesenchymal markers in ECs exposed to disturbed shear stress, as well as present in ECs overlying human atherosclerotic plaques (*10*). Consistent with reduced EndMT, Alk5 knockdown cells also showed decreased levels of *Notch3*, *fibronectin*, and *N-cadherin* (Fig. 1D). Furthermore, consistent with previous reports showing that shear stress induces only partial EndMT (*10*), we found that *VE-cadherin* (Vascular endothelial-cadherin) and *PECAM-1* (Platelet and endothelial cell adhesion molecule-1) were not reduced in ECs exposed to oscillatory shear stress even in Alk5-expressing cells; in contrast, eNOS (endothelial Nitric Oxide Synthase) was reduced in Alk5-control ECs compared to Alk5 knockdown ECs after exposure to shear stress (fig. S4). Together, these data provide several independent lines of evidence that support a role for endothelial Alk5 in flow-induced EndMT.

### Shc associates with Alk5 in response to shear stress and mediates mechanosensitive EndMT in vitro and in vivo

**Fig. 2:**
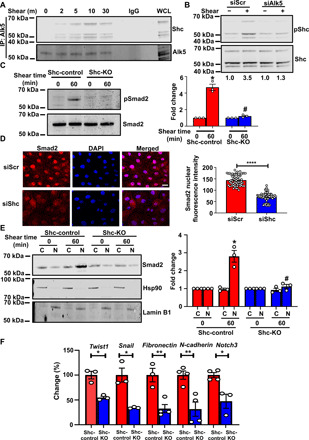
Shc associates with Alk5 and promotes shear stress–induced EndMT. (**A**) Coimmunoprecipitation of Alk5 with Shc after shear stress. *n* = 3. IgG, immunoglobulin G. WCL, whole cell lysate. (**B**) Scr or *Alk5* siRNA–transfected ECs were exposed to chronic oscillatory shear stress; Shc was immunoprecipitated and its Y^239,240^ phosphorylation was assayed. *n* = 3. (**C**) ECs from Shc-Control and Shc-KO mice were exposed to shear stress, and phosphorylation of Smad2 was determined. (**D**) Scr or *Shc* siRNA–transfected BAECs were exposed to shear stress; nuclear translocation of Smad2 was determined by immunofluorescence staining and quantified as mean fluorescence intensity on ImageJ. (**E**) ECs from Shc-control and Shc-KO mice were exposed to shear stress and separated into cytosolic and nuclear fractions. Western blots, performed using antibodies against the cytoplasmic protein Hsp90 and nuclear protein Lamin B1, indicated that the extracts are virtually free from cross-contamination. Hsp90 served as the loading control for cytoplasmic extracts and Lamin B1 for nuclear extracts. (**F**) ECs from Shc-Control and Shc-KO mice were exposed to chronic shear stress; qPCR was performed to quantify the expression of the EndMT markers *Twist1, Snail*, *fibronectin*, *N-cadherin*, and *Notch3*. Scale bar, 20 μm; *n* = 4. Data are presented as means ± SEM. *P* values were obtained by two-tailed Student’s *t* tests using GraphPad Prism. *P < 0.05; **P < 0.01, ****P < 0.001, and #*P* < 0.05 relative to the respective shear time point of Scr siRNA.

Having demonstrated that Alk5 is required for EndMT induced by shear stress, we next addressed the molecular mechanisms involved. Given that TGFβ recruits Shc to the Alk5-TGFβR2 complex (*25*) and loss of Shc ameliorates shear stress–induced endothelial activation (*22*), we examined whether Alk5 associates with Shc in response to shear stress. Immunoprecipitation of endogenous Alk5 from ECs exposed to acute shear stress brought down Shc; this association was specific as it was not observed when we used a nonspecific immunoglobulin G for immunoprecipitation (Fig. 2A). These results therefore provide evidence for an enhanced association between Shc and Alk5 upon exposure to shear stress and raised the possibility that Shc is part of the Alk5-dependent pathway that leads to EndMT.

To test whether Shc is activated downstream of Alk5, we subjected control and Alk siRNA–transfected ECs to shear stress and examined phosphorylation of Shc at Tyr^239/240^. Our data showed that while shear stress induces increased phosphorylation of Shc in control ECs, Alk5-depleted ECs display decreased phosphorylation (Fig. 2B). We then tested whether Shc is required for EndMT in vitro and in vivo. We subjected ECs isolated from Shc-control and Shc-knockout (KO) mice (fig. S1, B and C) (*23*) to acute and chronic oscillatory shear stress and examined phosphorylation of Smad2 (Fig. 2C and fig. S5A). In complementary experiments, we also examined nuclear translocation of total Smad2 in bovine aortic ECs (BAECs) transfected with Scr (scrambled) or Shc siRNAs (fig. S1D). ECs that lacked Shc displayed reduced nuclear translocation of Smad2 as assessed by both confocal microscopy and cellular fractionation studies compared to Shc-expressing cells (Fig. 2, D and E, and fig. 5B). We also examined up-regulation of EndMT markers in response to chronic oscillatory shear stress. Compared with controls, Shc KO cells showed significantly less mRNA expression of the EndMT transcription factors *Twist1* and *Snail*; reduced levels of *Notch3*, *fibronectin*, and *N-cadherin*; and increased levels of *eNOS* (Fig. 2F and fig. S6). In summary, these data support a role for endothelial Shc in flow-induced EndMT in vitro and show that loss of Shc phenocopies loss of Alk5.

**Fig. 3:**
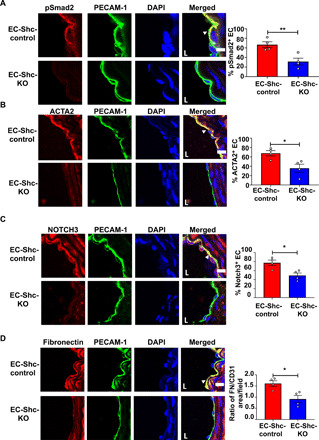
Shc mediates EndMT in disturbed shear stress regions in vivo. (**A** to **D**) Sections of the LCA from mice that underwent partial carotid ligation in the LCA, followed by 3 weeks of high-fat diet feeding, were immunostained with the phosphorylated form of the EndMT intermediary Smad2 and EndMT markers ACTA2, Notch3, and fibronectin. Staining was also performed with PECAM-1 and DAPI to identify the endothelium and nuclei, respectively. Positive cells (defined as coexpressing PECAM-1 and the respective EndMT marker) were quantified and shown by arrowheads. Scale bars, 10 μm; *n* = 4 EC-Shc-control mice and 4 EC-Shc-KO mice. Data are presented as means ± SEM. *P* values were obtained using two-tailed Student’s *t* tests using GraphPad Prism. *P < 0.05; **P < 0.01.

To complement these in vitro studies, we investigated the effects of Shc in vivo. On the basis of recent work highlighting the importance of EndMT in atherosclerosis (*9*, *10*, *13*, *14*, *18*), we sought to determine the role of endothelial Shc in both EndMT and atherosclerosis. To this end, Shc-floxed and endothelial-specific Shc-KO mice were crossed into the hypercholesterolaemic apolipoprotein E–deficient (ApoE^−/−^) background to generate EC-Shc-control and EC-Shc-KO. We confirmed deletion of Shc from ECs by analyzing Shc mRNA and protein levels of ECs isolated from mouse aorta (fig. S1, B and C). We also controlled for potential Cre toxicity by showing that injection of tamoxifen in Cre-positive animals in which Shc is not floxed does not affect flow responses in the aortic arch (fig. S7). To specifically test the role of Shc in a model of oscillatory and disturbed flow in vivo, we used the partial carotid ligation model; in this model, three of the four caudal branches of the left carotid artery (LCA) are ligated, resulting in substantial reduction in blood flow velocity and change of flow pattern from uniform to oscillatory in the LCA (fig. S8) (*26*). Corroborating in vitro findings, oscillatory flow regions of EC-Shc-control mice stained strongly for pSmad2, while these were reduced in EC-Shc-KO mice (Fig. 3A). We also examined the subcellular localization of total Smad2 and found that EC-Shc-control mice displayed higher levels of nuclear Smad2 compared to EC-Shc-KO mice (fig. S9). In agreement with activation of the EndMT program, there was also an increase in protein expression of smooth muscle cell markers (Notch3 and ACTA2) and the extracellular cell matrix molecule fibronectin in EC-Shc-control compared to EC-Shc-KO mice (Fig. 3, B to D). The right carotid arteries (RCAs; which are exposed to laminar flow) did not show up-regulation of EndMT markers (fig. S10). Together with the above studies, these data indicate that Shc couples oscillatory shear stress to activation of the EndMT program.

### Shc deletion reduces atherosclerotic plaque formation in areas of disturbed shear stress

**Fig. 4:**
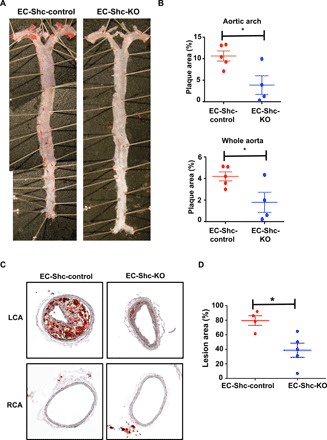
Shc regulates atherosclerotic plaque formation in areas of atheroprone shear stress. (**A**) Representative en face preparations of whole aortas showing atherosclerosis in EC-Shc-control and EC-Shc-KO mice after 8 weeks of high-fat diet feeding, visualized by Oil Red O staining. Photo credit: Jianhua Huang, University of North Carolina at Chapel Hill. (**B**) Quantification of the percentage plaque area in whole aortas and aortic arches from EC-Shc-control and EC-Shc-KO mice. *n* = 5 EC-Shc-control mice and 4 EC-Shc-KO mice. (**C**) Representative images of plaque deposition in the LCA after partial carotid ligation and 3 weeks of high-fat diet feeding in EC-Shc-control and EC-Shc-KO mice. The RCA served as an unoperated control. (**D**) Quantification of the percentage lesion area in the LCAs from EC-Shc-control and EC-Shc-KO mice after partial carotid ligation and high-fat diet feeding. *n* = 4 EC-Shc-control mice and 5 EC-Shc-KO mice. Data are presented as means ± SEM. *P* values were obtained using two-tailed Student’s *t* tests using GraphPad Prism. *P < 0.05.

Having shown a role for Shc in flow-induced EndMT, we sought to determine whether atherosclerosis was also affected by deletion of Shc. We assessed this in two murine models of atherosclerosis. After high-fat diet feeding to induce atherosclerotic lesions, en face analysis of Oil Red O–stained aortas was carried out and calculated as percentage of lesion area compared with total surface area for both the whole aorta and the aortic arch. EC-Shc-control mice displayed lesions particularly in the aortic arch, a site of disturbed blood flow and therefore highly susceptible to plaque deposits (Fig. 4, A and B). Despite comparable serum cholesterol levels (1681 versus 1745 mg/dl), the aortic arches from EC-Shc-KO mice showed negligible atherosclerotic lesions. The decrease in plaque burden in EC-Shc-KO mice was also true for the whole aorta.

In complementary experiments, we also tested the contribution of endothelial Shc to flow-induced atherosclerosis using the partial carotid ligation model. The distinct advantage of this model is that it provides a direct opportunity to study the role of individual mechanotransduction genes in a low/oscillatory blood flow setting and atherogenesis, as the disturbance of flow is acute and plaque progression occurs rapidly in an otherwise atheroresistant vessel. Partial ligation of the LCA followed by 3 weeks of high-fat diet feeding led to extensive circumferential plaque deposition in the carotid arteries of EC-Shc-control mice. In contrast, the carotid arteries of EC-Shc-KO mice appeared to be resistant to plaque development (Fig. 4, C and D). In either of the mice, the RCA that was not operated upon and therefore exposed only to laminar blood flow did not show any evidence of atherosclerotic deposition (Fig. 4C).

### Alk5 is a unique and sufficient mechanosensor for EndMT

**Fig. 5:**
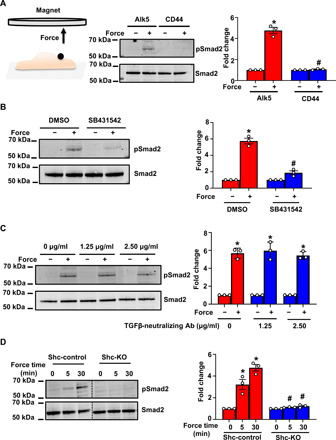
Force on Alk5 specifically induces mechano-EndMT via Shc. (**A**) Mouse ECs were incubated with anti-Alk5 or CD44 (negative control) antibody–coated beads and subjected to force (10 pN). Phosphorylation of Smad2 was determined by Western blotting and quantified using Image Studio Lite v.5.2. *n* = 3 (**B** and **C**) Mouse ECs were treated with the (B) Alk5 kinase inhibitor SB431542 or (C) varying concentrations of a TGFβ-neutralizing antibody (Ab), incubated with anti-Alk5–coated beads and subjected to force before analysis of the phosphorylation of Smad2. *n* = 3. Data are presented as means ± SEM. *P* values were obtained using two-tailed Student’s *t* test using GraphPad Prism. DMSO, dimethyl sulfoxide. (**D**) Shc-control and Shc-KO ECs were incubated with anti-Alk5–coated beads and subjected to force application before analysis of the phosphorylation of Smad2. *n* = 3. Data are presented as means ± SEM. *P* values were obtained using two-tailed Student’s *t* test using GraphPad Prism. Phosphorylated proteins are indicated by “p-.” *P < 0.05 relative to the no-force condition, and #*P* < 0.05 relative to the respective force application time point with Alk5.

Our data thus far identify an Alk5-Shc pathway that regulates flow-induced EndMT and atherosclerosis. Does Alk5 sense flow or is it simply a player in mechanochemical cascades? The requirement for Alk5 in activation of EndMT in response to shear stress in vitro (Fig. 1) prompted us to investigate whether Alk5 acts as a mechanoreceptor; to test this, we considered whether mechanical force on Alk5 can induce signaling relevant to EndMT. We used a magnetic system to stimulate magnetic beads coated with anti-Alk5 antibody and measured activation of Smad2. These experiments were performed in the presence of serum-free media, as fetal bovine serum (FBS) is known to contain significant amounts of TGFs and bone morphogenetic proteins that could activate the receptor. As shown in Fig. 5A, tensional force application on Alk5 induced robust phosphorylation of Smad2; this was specific to Alk5, as cells incubated with the transmembrane receptor CD44 did not respond to force. To investigate specificity of this response, we used an Alk5 inhibitor (SB431542), which interferes with receptor activation and has been shown to inhibit shear stress–induced Smad2/3 activation (*17*). Pretreatment of ECs with the Alk5 inhibitor abolished the increase in Smad2 phosphorylation in response to tensional forces on Alk5 (Fig. 5B), indicating that the kinase activity of Alk5 is required for downstream force-induced Smad signaling. In addition, to determine whether the Alk5 force response is independent of its ligand, we used a TGFβ-neutralizing antibody. We first validated that the neutralizing antibody treatment works by verifying that it blocks TGFβ-induced Smad2 phosphorylation (fig. S11). Treatment of cells before and during force application with the neutralizing antibody did not alter the force response, suggesting that force on Alk5 activates Smad2 in a ligand-independent manner (Fig. 5C). Last, to determine whether Shc mediates the mechanical response of Alk5 to force, we examined the same responses in Shc KO cells. We observed that cells lacking expression of Shc failed to respond to mechanical stimulation of Alk5 (Fig. 5D), thus strengthening the connection between force, Alk5, and Shc. Overall, these results show for the first time that mechanical force on Alk5 can induce activation of Smad2 in a ligand-independent manner and that this is dependent on Shc.

**Fig. 6:**
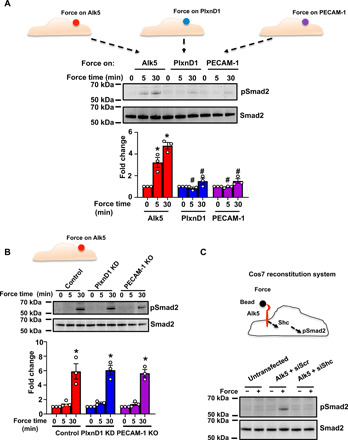
Alk5 is unique and sufficient to induce EndMT signaling in response to force. (**A**) Mouse ECs were incubated with anti-Alk5, anti-PLXND1, or anti–PECAM-1 antibody–coated beads and subjected to force (10 pN) for the indicated time periods. Phosphorylation of Smad2 was determined by Western blotting and quantified using Image Studio Lite v.5.2. *n* = 3. (**B**) Mouse ECs transfected with control siRNA or siRNA against *PlxnD1*, or PECAM-1 KO cells were incubated with anti-Alk5–coated beads and subjected to force for the indicated times before analysis of the phosphorylation of Smad2. *n* = 3. KD, knockdown. (**C**) Cos7 cells were left untransfected or transfected with pcDNA-Alk5 and either Scr or Shc siRNA. The cells were incubated with anti-Alk5–coated beads and subjected to force before analysis of the phosphorylation of Smad2 (*n* = 3). *P < 0.05 relative to the no-force condition, and #*P* < 0.05 relative to the respective force application time point with Alk5.

We have previously shown that the junctional cell adhesion molecule PECAM-1 and, more recently, the cell guidance receptor PlxnD1 sense and respond to mechanical force by eliciting activation of mechanosensitive pathways (*27*–*29*). To investigate whether the force-induced activation of Smad2 is a unique response to Alk5 or shared by other mechanoreceptors, we applied tensional forces on PECAM-1 or PlxnD1 and examined phosphorylation of Smad2. Consistent with Fig. 5A, force on Alk5 induced Smad2 phosphorylation (Fig. 6A); in contrast, force application on either PECAM-1 or PlxnD1 did not elicit phosphorylation of Smad2 (Fig. 6A), suggesting that activation of this pathway occurs specifically downstream of Alk5.

We then investigated a possible interrelationship or synergy between these mechanosensors. To this end, we examined whether Alk5-dependent mechanoactivation of Smad2 requires the presence of PECAM-1 or PlxnD1. We performed the same magnetic force experiments on Alk5 in ECs in which PlxnD1 had been knocked down (fig. S1E) or in PECAM-1 KO ECs (Fig. 6B). Our results demonstrate that force on Alk5 induces the Smad2 mechanoresponse even in cells that lack expression of PECAM-1 or PlxnD1, suggesting that Alk5 not only is unique in activating mechano-EndMT signals but also it does so independently of the other mechanosensors.

The unique ability of Alk5 to induce Smad2 phosphorylation in response to force prompted us to determine whether Alk5 is sufficient to confer mechanoactivation of this pathway in a heterologous cell line. For these experiments, we used Cos7 cells that we transfected with a plasmid construct expressing Alk5 (Fig. 6C). These cells do not express Alk5 but do express endogenous Shc and Smad2 and, thus, constitute an ideal system to monitor both mechanical responses due to Alk5 and the role of Shc in this system by transfecting with Scr or Shc siRNAs (fig. S1F). Cos7 cells were either untransfected or transfected with plasmid expressing Alk5 and Scr or Shc siRNA before force application on Alk5 using paramagnetic beads. Cells expressing Alk5 and transfected with Scr siRNA showed activation of Smad2 in response to force. In contrast, cells expressing Alk5 and transfected with Shc siRNAs did not activate Smad2, demonstrating that force on Alk5 elicits Smad2 activation in a heterologous cell line and that this activation is dependent on Shc (Fig. 6C). Consistent with data shown in Fig. 6A, expression of either PlxnD1 or PECAM-1 in Cos7 cells and force application on each respective mechanosensor was not enough to induce phosphorylation of Smad2 (fig. S12). Together, these data unambiguously show that Alk5 operates as a unique and sufficient force sensor for activation of the Smad2 pathway in a Shc-dependent manner.

## DISCUSSION

There is a growing appreciation of the importance of disturbed shear stress in EndMT and ensuing plaque growth in atherosclerosis. Although the TGFβ-Smad2/3 pathway plays a central role in EndMT, the mechanoreceptor responsible for activation of EndMT in atherosclerosis-prone regions of arteries was elusive. In this study, we identify the mechanoreceptor that uniquely drives mechano-EndMT in ECs and uncover a molecular mechanism directly connecting force to the activation of biochemical signaling for EndMT. We show that depletion of Alk5 abrogates shear stress–induced EndMT signaling, including activation of Smad2 and downstream up-regulation of mesenchymal and ECM genes. Tensional force and reconstitution experiments unveil a mechanosensory function for Alk5 in EndMT signaling that is unique to Alk5 and independent of other known mechanosensors. Previous studies have pointed toward a role for the TGFβ-Alk5 pathway in shear stress responses (*15*–*17*). In contrast, our study demonstrates that mechanical force activates Alk5 independent of its ligand, as force on Alk5 elicits EndMT signals in a TGFβ-independent manner.

How does Alk5 regulate flow-dependent EndMT? We identify the adaptor protein Shc as a critical mediator of the Alk5 force-response and reveal that genetic targeting of endothelial Shc reduces EndMT and atherosclerosis in areas of disturbed flow. Although our study shows a reduction in atherosclerotic burden in EC-Shc-KO mice, we cannot definitively say, at this point, that this is the result of lower EndMT (which would be strengthened by the use of lineage tracing approaches). Furthermore, a role for Shc in physiological laminar shear stress responses has not been determined yet but is the subject of current intense investigation.

By unveiling a specific pathway activated as a result of force application on Alk5, our study makes a direct link between mechanical force, EndMT, and cell plasticity. EndMT is a crucial process during development of cardiac atrioventricular valves but is also a recently described feature of vascular pathologies characterized by abnormal shear stress. Although the exact definition of EndMT (is it true transdifferentiation or vascular mimicry?) is a subject of intense debate in the literature, activation of ECs that includes expression of mesenchymal genes is clearly a pathologic response. Our discovery of a pathway that links mechanical forces to EndMT opens up previously unidentified avenues for therapeutic targeting of EndMT in diseases that form as a result of converging biomechanical and inflammatory factors.

## METHODS

### Experimental animals

All animal experiments were approved and authorized both by the University of Oxford Local Animals Ethics and Welfare Committee and by the Home Office, UK. The project license used in this work was P9133D191. Shc^fl/fl^ animals were obtained from K. Ravichandran (University of Virginia), and Cdh5-Cre mice were obtained from R. Adams (Max Planck Institute, Muenster). To obtain endothelial specific deletion of Shc, these mice were crossed with mice expressing either a constitutive or inducible Cre recombinase under the *Cadherin-5 Cre* or *Tie2 Cre* driver. Three consecutive intraperitoneal injections of tamoxifen (2 mg each) in adult animals (6 to 8 weeks of age) resulted in the deletion of endothelial Shc. Mice lacking endothelial Shc are designated as Shc^ECKO^. For atherosclerosis studies, the Shc^fl/fl^ or Shc^ECKO^ animals were crossed into the hypercholesterolemic ApoE^−/−^ background to generate EC-Shc-control and EC-Shc-KO mice. All mice used in this study were maintained on a C57BL/6J background. For en face immunofluorescent analysis, tissue was harvested 2 weeks after the last tamoxifen injection. All animals were housed in individually ventilated cages at 22°C, 56% relative humidity, and light/dark cycle of 12 hours/12 hours and fed on a standard chow diet (B&K Ltd., UK). For high-fat diet experiments carried out on animals in the hypercholesterolemic ApoE^−/−^ background, the animals were fed Western RD (P) VP 25-kGy diet containing 20% fat and 0.15% cholesterol [829108, SDS (Special Diets Services), UK] for 8 weeks. Water and food were available ad lib at all times.

### Inhibitors, antibodies, and other reagents

The antibodies used for Western blotting included phosphorylated (p)-Smad2 (S465/467, Cell Signaling Technology), Smad2 (Cell Signaling Technology), p-Shc (Y239,240, Cell Signaling Technology), Shc (Abcam), PlxnD1 (Thermo Fisher Scientific), PECAM-1 (Santa Cruz Biotechnology), Alk5 (Sigma-Aldrich for biochemical assays and R&D for tensional force application), TGFβ1 to TGFβ3 neutralization antibody (R&D), Lamin B1 (Abcam), and heat shock protein 90 (Hsp90; Cell Signaling Technology). The inhibitor SB431542 (Sigma-Aldrich) was used at a concentration of 10 μM. Recombinant mouse TGFβ2 was purchased from R&D Systems (Bio-Techne) and used at 5 ng/ml.

### Partial carotid ligation

Partial carotid ligation was performed as previously described (*26*, *30*). Briefly, female mice were ligated between 6 and 8 weeks of age. All mice were fed a chow diet and water ad lib until partial ligation. Three of the four caudal branches of the LCA (left external carotid, internal carotid, and occipital artery) were ligated while the superior thyroid artery was left untouched. Mice were fed a Western diet immediately after partial carotid ligation until they were euthanized 3 weeks later, and their carotid arteries were harvested.

### Genotyping

Genotyping was determined by polymerase chain reaction (PCR) analysis of DNA in ear notches, collected for identifying the animals, using the Phire Tissue Kit (F140-WH, Thermo Fisher Scientific).

### En face preparations

Animals were induced a terminal general anesthesia with isoflurane followed by exsanguination and perfusion fixation with 4% paraformaldehyde. The entire length of the aorta was dissected out, and the surrounding connective tissue and adventitial fat were removed. The aorta was fixed in 4% paraformaldehyde and stored at 4°C in phosphate-buffered saline (PBS) until staining. Atheroprone areas from the inner curvature of the aortic arch were isolated and processed for immunofluorescent studies.

### Oil Red O staining for atherosclerosis study

Fixed aortas were rinsed in absolute propylene glycol and stained with Oil Red O (01516, Sigma-Aldrich). After washing in 85% propylene glycol solution and distilled water, the aortas were opened longitudinally to the iliac bifurcation, and a coverslip was placed to flatten down the aorta with endothelial surface facing upward. Images were acquired using Olympus SZX7 fitted with a 1× lens, and image processing was performed using Image-Pro (Media Cybernetics). The plaque area was quantified as a percentage of the area of both the total aorta and the aortic arch. For partial carotid ligation studies, OCT (optimal cutting temperature compound)–embedded LCA (ligated) and RCA (sham) sections were stained with Oil Red O dissolved in isopropanol.

### Lipid profile analysis

Blood was sampled by cardiac puncture under terminal general anesthesia in plasma collection tubes. Plasma samples were shipped to MRC Harwell, where they were analyzed for total cholesterol on an automated AU680 Clinical Chemistry Analyzer.

### Cell culture and shear stress

Mouse lung ECs were isolated from Shc^fl/fl^ mice and Shc^ECKO^ mice and maintained in EGM2 growth medium (Lonza) supplemented with 10% FBS. PECAM-1 KO mouse cells were cultured as previously described (*31*). All cell types were maintained at 37°C in 5% CO_2_ in a humidified incubator. Cells were subjected to shear stress either using a parallel plate chamber (*31*), a cone-and-plate viscometer as previously described (*32*). BAECs were subjected to oscillatory (±4 dynes/cm^2^, 1 Hz) flow in the Ibidi Pump System (Ibidi) or kept under static conditions for 24 hours. siRNA reverse transfections of the genes of interest in mouse ECs and BAECs were performed using the Lipofectamine RNAimax Reagent (Invitrogen). siRNAs used in this study were from Dharmacon and are described below.

#### Alk5

The *Alk5* SMARTpool consisted of GCACCACCUUAAAAGAUUU, UCGAUUACUUGAAUAGAUA*, CGUUUAGCCUGAGUUUAUU*, and GGGAGAUGCUUAAAUUUCA. siRNAs marked with * were used for single-siRNA experiments.

#### Shc

The *Shc* SMARTpool consisted of GGAGUAACCUGAAAUUUGC, CCAUCAGUCUGGUGUGUGA, GUUUCCUACUUGGUUCGGU, and CAAUCUAUCUCAUUUGCAU.

#### PlxnD1

The *Plxnd1* SMARTpool consisted of GUAUCGACCACAGAUCAUG, CGUGGACCUUGAAUGGUUU, CUAUUAUAAACAGAUCCAA, and CCAACAAGCUUCUGUACGC.

Plasmids encoding *Alk5, PlxnD1* and *PECAM-1* were used for over-expression in Cos7 cells. Transfections of these plasmids were performed with Lipofectamine 2000 (Invitrogen) according to the manufacturer’s instructions.

### RNA extraction and quantitative PCR

Total RNA extraction was performed from cells using the RNeasy Plus mini kit (Qiagen) with an additional genomic DNA wipeout step. Reverse transcription was performed using the Superscript III cDNA synthesis kit. Quantitative real-time PCR was performed in triplicate with SYBR green and CFX96TM real-time system. Thermocycling conditions were 95°C for 3 min, followed by 40 cycles of 95°C for 15 s and 60°C for 45 s. Gene expression was normalized to the constitutively expressed housekeeping gene 18*S* rRNA, and relative expression was calculated and plotted using the ΔΔCt method. Primer sequences used were as follows: N-cadherin: 5′-ATGGATGAAACGGCGGGATA-3′ (forward) and 5′-TGATTCCCACGGGCTTGATG-3′ (reverse); fibronectin: 5′-CCAGCCGCAAAGAGTCTACA-3′ (forward) and 5′-GGGTTTCCACGTCTCACCAT-3′ (reverse); Notch3: 5′-GTCTGGATGGAAGCCCATGT-3′ (forward) and 5′-CTTGGAAGCCACGGAGACAA-3′ (reverse); Twist1: 5′-GCCGGAGACCTAGATGTCATTG-3′ (forward) and 5′- CCACGCCCTGATTCTTGTGA −3’ (reverse); Snail: 5′-TCTGCACGACCTGTGGAAAG-3′ (forward) and 5′-CACATCCGAGTGGGTTTGGA-3’ (reverse); eNOS: 5′-GAGAGCGAGCTGGTGTTTGG-3′ (forward) and 5′-GGGAACACTGTGATGGCTGAA-3′ (reverse); VE-cadherin: 5′-CTAACCGTGGGTGTGTGCA-3′ (forward) and 5′-TCCAGCGCACTCTTGCTATG-3′ (reverse); PECAM-1: 5′-CACACCGAGAGCTACGTCAT-3′ (forward) and 5′-CGCCTTCTGTCACCTCCTTT-3′ (reverse); Shc: 5′-CTCAGGATGTCATCAGCACCAT-3′ (forward) and 5′-TCCCAAGCTGAGCCATCAAAG-3′ (reverse); and 18*S* rRNA: 5′-AGGAATTGACGGAAGGGCACCA-3′ (forward) and 5’-GTGCAGCCCCGGACATCTAAG-3′ (reverse).

### Immunofluorescence

Tissue and cell permeabilization was performed by incubation with 0.5% Triton X-100 overnight and 0.2% Triton X-100, respectively, and blocked with 10% normal goat serum/1% BSA. Frozen sections of partial carotid ligation tissues were incubated with phospho-Smad2 (S451/467) (3108S, Cell Signaling Technology), NOTCH3 (ab23426, Abcam), fibronectin (HFN7.1, DSHB), ACTA2 (A5228, Sigma-Aldrich), and PECAM-1 (553369, BD Pharmingen) followed by Alexa Fluor 488 or 568, respectively. Inner curvatures of the aortic arch were incubated with primary antibodies ICAM-1 (YN 1/1.7.4) (116101, BioLegend) and MCP-1 (ab7202, Abcam) before incubation with Alexa Fluor 568–conjugated secondary antibodies (1:100, Invitrogen). Cells subjected to flow were incubated at 4°C overnight with total Smad2 (5339S, Cell Signaling Technology) followed by Alexa Fluor 568–conjugated secondary antibody at room temperature and 4′,6-diamidino-2-phenylindole (Invitrogen). Cells and tissues were mounted en face with Prolong Gold Antifade mountant (Invitrogen) for confocal imaging using Olympus FluoView 3000.

### Image analysis

Images were converted to 8-bit, and the threshold was adjusted. Nuclear Smad2 translocation was computed by analyzing the mean fluorescent intensity of the stain present in the nucleus using ImageJ software (Analyze ➔ Analyze particles). For tissue sections, positive cells were defined as coexpressing PECAM-1 and the respective EndMT marker. All images were obtained and quantified in a blinded fashion.

### Coimmunoprecipitation and Western blotting

For signaling studies, cells were lysed in radioimmunoprecipitation assay buffer (Sigma-Aldrich) supplemented with protease and phosphatase inhibitor cocktails. For subcellular fractionation studies, the Nuclear Extraction Kit (Abcam) was used as per the manufacturer’s instructions. For coimmunoprecipitation studies, cells were collected in lysis buffer as previously described (*22*, *29*) and supplemented with protease and phosphatase inhibitor cocktail tablets. Lysates were precleared with 10 μl of protein A/G plus sepharose beads (Santa Cruz Biotechnology) for 1 hour at 4°C. The precleared lysates were then incubated with 20 μl of protein A/G plus sepharose beads, which had previously been coupled with the appropriate primary antibody for 2 hours at 4°C on an orbital shaker. The beads were washed three times with lysis buffer supplemented with protease and phosphatase inhibitors. The immunoprecipitation complexes were eluted from the beads by boiling in 2× SDS buffer for 5 min.

For all Western blotting analyses, protein lysates and coimmunoprecipitation complexes were resolved on a 4 to 12% gradient gel with the appropriate primary antibodies and IRDye-conjugated anti-mouse, anti-goat, or anti-rabbit secondary antibodies, as appropriate. Images were acquired on a LICOR Odyssey infrared scanner. Densitometric quantification of bands was performed using ImageStudio software (LICOR Biosciences).

### Tensional force application/magnetic tweezer system

Tosyl-activated paramagnetic beads (4.5 μm) were washed with PBS and coated with an antibody against the extracellular domain of Alk5 (R&D), PLXND1 (Santa Cruz Biotechnology), PECAM-1 (a gift from P. Newman), or CD44 (clone 5D2-27 from the Developmental Studies Hybridoma Bank, USA). Beads were quenched in 0.2 M tris (pH 7.4) before use to eliminate any remaining tosyl groups. ECs were incubated with the beads (and inhibitor or blocking antibody, if appropriate) for 30 min in serum-free M199 before force application using a permanent ceramic magnet for another 30 min at 37°C. For all experiments, the magnet was placed parallel to the cell surface and at a distance of 0.6 cm, so that the force experienced by a single bead was approximately 10 pN. Following tensional force application, the cells were washed with PBS, snap-frozen on dry ice, and immediately lysed. To analyze the phosphorylation of Smad2, the lysates were immunoblotted with a primary antibody against pSmad2.

### Statistics

Data are presented as means ± SEM. All experiments were performed at least three times independently. Statistical significance was tested by using unpaired Student’s *t* tests as indicated in Results. Data were tested for normality using the Shapiro-Wilk test and equality of variance using the Levene test, where necessary data were log-transformed before being analyzed for statistical significance. All image analyses were performed by operators who were blinded to the treatments administered.

## SUPPLEMENTARY MATERIALS

Supplementary material for this article is available at http://advances.sciencemag.org/cgi/content/full/7/28/eabg5060/DC1

## Appendix

View/request a protocol for this paper from *Bio-protocol*.
